# A double-staining automated flow cytometry method for real-time monitoring of bacteria in continuous bioreactors

**DOI:** 10.1038/s41540-026-00694-3

**Published:** 2026-04-01

**Authors:** Juan López-Gálvez, Erik Schönfelder, Hanna Mayer, Konstanze Schiessl, Marisa O. D. Silva, Hauke Harms, Susann Müller

**Affiliations:** 1 Department of Applied Microbial Ecology, Helmholtz-Centre for Environmental Research – UFZ, Leipzig, Germany; 2 onCyt Microbiology AG, Otelfingen, Switzerland

**Keywords:** Biological techniques, Biotechnology, Microbiology

## Abstract

In biotechnological processes, cell density and physiology are critical parameters for controlling the feed rate, harvest time, and process performance. We developed an automated flow cytometry approach that enables continuous, real-time (fully automated, hourly) monitoring of bacterial populations in continuous bioreactors. The method employed a double-staining protocol that combined DAPI to assess total DNA content and Alexa Fluor 488-EdU via Click-iT technology to identify the proportions of cells undergoing active DNA replication through EdU incorporation. The integrated workflow included fixation, permeabilization, staining, and measurement steps and was applied to three Gram-negative strains: *Bradyrhizobium* sp., *Escherichia coli*, and *Stenotrophomonas rhizophila*. Automated analysis captured growth dynamics and cell cycle progression, providing insights into population behavior under different dilution rates. In this study, automated on-line sampling enabled hourly flow cytometry measurements of cell concentration and physiological indicators during continuous cultivation, supporting real-time monitoring and control in industrial biotechnology.

## Introduction

Cell density is a fundamental parameter of cell cultivation and affects feed rate, harvest time, and product yield^1^. Monitoring and adjusting cell density are crucial for optimizing bioprocesses and achieving desired production outcomes^2^. In addition to absolute cell density, the dynamics of biomass and process performance depend on the physiological state of the population, therefore, we use automated flow cytometry to quantify cell concentration directly and to complement it with single-cell indicators of growth and replication activity, like mean DAPI fluorescence and DNA replication activity. Traditionally, biomass has been measured indirectly through optical density (OD), capacitance-based biomass sensors, or dry cell weight measurements. The measurement of OD can be limited by the composition of the medium (e.g., opaque media) and does not differentiate between morphologies related to cell state. Capacitance measurements, on the other hand, are sensitive to particles, cell fragments, and high cell densities. Small cells may fall below the detection limit. Traditional dry weight cell measurements require manual sampling and handling, which makes them time-consuming, prone to operator-dependent variability, and reduced reproducibility due to human error. By automating sampling and staining, the workflow enables consistent hourly measurements, which is a higher measurement frequency than conventional manual flow cytometry or dry cell weight-based biomass measurements and was sufficient to resolve the physiological changes observed during continuous cultivation in this study. Another common method is the measurement of OD. OD provides a continuous, online or at-line estimate of turbidity that is proportional to biomass after path-length and strain-specific calibration. As a bulk optical signal, OD reflects particle/light-scattering properties and can be influenced by cell size, morphology, clumping, and medium composition, yet it remains a robust and practical real-time proxy in many fermentations. However, it cannot resolve population heterogeneity and it is an indirect measurement of bacterial concentration^3^. Advanced sensor technologies that offer continuous, real-time data by directly measuring spectroscopic characteristics^4,5^ can capture the molecular fingerprints of the population and the surrounding medium, linking the biomass to metabolic activity. However, they are not yet applicable for routine use once the required instrumentation and expertise are available. In routine settings, single-cell-based analysis tools, such as low-cost automated counters, are used to estimate cell numbers, but they do not report on cell physiology. More advanced devices support fluorescence-based live/dead cell readouts^6^. This situation underscores the need for monitoring methods that are both more insightful and more operational.

Automated flow cytometry has emerged as a powerful tool for monitoring microbial cell states and physiology. The technique offers rapid, quantitative, and cell-functional insights into bioreactor dynamics, which are essential for optimizing bioprocesses^7,8^. Conventional off-line flow cytometry has been used for decades in biotechnology to measure variations in cell concentration and distinguish different cell states based on morphology and function within productive microbial processes^9^. For example, conventional flow cytometry has been used to optimize the production of recombinant *Escherichia coli* fed-batch fermentations at high cell densities, including production of recombinant antibody fragments (Fab) and related process optimization using reporter strains^10,11^ as well as in electricity generation processes in microbial fuel cells, where an understanding of microbial community structure is vital for process optimization^12^. Based on experience with conventional flow cytometry, automated flow cytometry is primarily used to monitor microorganisms in technical environments. Multiplexable automated setups have been employed to track green fluorescent protein (GFP) reporter expression and/or propidium iodide uptake, which enabled a detailed characterization of stress dynamics under changing process conditions^13^. The technology has also been used to quantify phenotypic variability during scaling up of continuous and fed-batch processes, where changes in operational parameters altered cell-to-cell heterogeneity^14^. Another example is the characterization of oleaginous yeast cells, in which changes in cell-physiological states served to operate fermentation processes^15^. Currently, a concept of automated and reactive flow cytometry has been introduced, where online population data are directly coupled to bioreactor control to trigger medium pulses or environmental switches, for example, in segregostat-like operation modes, to stabilize co-cultures or dynamically control gene circuit expression in synthetic biotechnology^8^. Additionally, automated cytometry has been routinely used beyond managed environments and is essential for analyzing the microbiome of drinking water in order to assess its quality and microbial safety^16–18^. Automated flow cytometry has also been used effectively in the characterization of marine prokaryote communities in open water systems^19–21^. These approaches involve trade-offs between sampling frequency, information content, and practical effort. OD and capacitance are broadly accessible but provide limited physiological resolution and can be biased by morphology. Spectroscopic and off-gas methods can be non-invasive but are often calibration- and process-specific. Flow cytometry provides single-cell physiological information and population structure, but requires specialized instrumentation and expertise.

The physiological states of cells are largely governed by their growth regime. These differences can profoundly impact cellular productivity in bioreactor setups^22,23^. In a previous study, we demonstrated the use of automated flow cytometry to analyze chromosome numbers in microbial cells to measure cell growth. The combination of an OC-300 cell sampler (onCyt Microbiology, Zürich, Switzerland), which can automatically collect and stain cells, and a CytoFLEX S flow cytometer (Beckman Coulter, Brea, CA, USA) has effectively delivered stable cell counts and facilitated the monitoring of bacterial growth dynamics within generation times. This high-resolution monitoring enabled for real-time detection of shifts in culture composition^7^. Building on this, we expanded our analysis in this study to include detecting cell cycle proliferation dynamics. To this end, we used two complementary dyes that enabled us to monitor the total cellular DNA and the fraction of cells undergoing DNA synthesis in real-time in an automated on-line workflow. The protocol eliminated the need for centrifugation steps, even though the cells were fixed, permeabilized, stained, and diluted, to facilitate online monitoring of growth activities in biotechnological systems.

DAPI (4′,6-diamidino-2-phenylindole) and Alexa Fluor 488 were used to simultaneously assess total DNA content and DNA synthesis. DAPI is an AT-specific DNA stain enabling for counting chromosome numbers of microbial cells^24^, whereas the Click-iT technology uses EdU (5-ethynyl-2′-deoxyuridine), a thymidine analog present in the culture medium. EdU is incorporated into newly synthesized DNA strands during replication^25^. After incorporation, the EdU residue undergoes a copper-catalyzed click chemistry reaction and binds covalently to an Alexa Fluor 488 dye, which can then be detected by flow cytometry^26^. The Alexa 488 Click-iT technology is best known for the analysis of human and mammalian cultures^27,28^, but EdU click labeling has also been adapted to a few bacterial systems, where it has been used to detect newly replicated DNA^26,29^. However, its use in applied bacterial biotechnology and bioprocess monitoring is not yet reported. In bacterial flow cytometry, DAPI fluorescence intensity is a validated quantitative marker of cellular DNA content, which scales with genome equivalents and distinguishes cell-cycle subpopulations that are consistent with bacterial replication physiology^24,30^. Instead, EdU incorporation directly marks ongoing DNA replication, conceptually analogous to the classical BrdU pulse labeling method, which was established in the 1980s for flow cytometric analysis of DNA synthesis in human cells^31^. The fraction of EdU-positive events provides an operational measure of the size of a replicating subpopulation^27,32,33^.

This study utilized three distinct Gram-negative strains: *Bradyrhizobium* sp., *Escherichia coli*, and *Stenotrophomonas rhizophila*. We applied the double-staining technique to these strains in a continuous bioreactor to test whether cell growth and heterogeneous DNA replication dynamics can be monitored and analyzed using online flow cytometry. Using the same automated workflow for all three species allowed us to assess its feasibility and robustness beyond a single model organism and to determine whether actively replicating bacteria can be detected in continuous cultures. We selected these safe, nonpathogenic strains with complementary relevance. *E. coli* and *S. rhizophila* are both included in the defined bacterial mock community used for cytometry standardization^7,34^. *E. coli* is also the most commonly used organism in methodological studies, providing a well-characterized benchmark. *S. rhizophila* and *Bradyrhizobium* sp. represent plant-associated bacteria important for soil and plant health; *Bradyrhizobium* strains are already biotechnologically produced and applied as commercial seed coatings and inoculants to support early crop establishment^35^, whereas *S. rhizophila* is being developed and tested as a plant growth-promoting and biocontrol agent in agricultural systems^36^. In this study, we establish and benchmark an automated on-line DAPI—Alexa 488-EdU flow cytometry workflow in continuous bioreactors to determine whether actively replicating subpopulations and growth dynamics can be robustly monitored across different Gram-negative bacteria.

## Results

We used OD and dissolved oxygen as bulk process indicators. To determine the effect of changes in growth rates on individual cell parameters, online flow cytometry was employed as a single-cell complement. Flow cytometry delivered cell counts and multi-parameter single-cell readouts as 2D dot plots revealing subpopulation structure (heterogeneity). The automated flow cytometric workflow was applied in two experimental formats. First, continuously operated 10 mL bioreactors were monitored under defined dilution rates to quantify time resolved changes in cell concentration and single-cell readouts. Second, during method development, 1 mL batch cultures in 24-well plates were used for method validation and for estimating strain-specific growth characteristics. Prior to DAPI staining, cells were sampled, fixed, and permeabilized as described in “Methods”, DAPI and Alexa 488-EdU double staining procedure for automated determination of cell concentration, mean DAPI-fluorescence intensity (FI), and DNA replication activity analysis. The per-cell DAPI-FI served as a proxy for DNA content and the average DAPI fluorescence intensity per cell reflects the average cellular DNA content. During periods of active DNA synthesis, cells increase their DNA content through chromosome replication, therefore an increase in average DAPI-FI indicates a shift toward higher DNA content consistent with ongoing DNA replication at the population level. The Alexa Fluor 488 label served as a marker for the EdU incorporation during DNA replication, which was performed simultaneously with the DAPI staining.

### Online cytometry as a sensor for bacterial growth by DNA content analysis

**Fig. 1:**
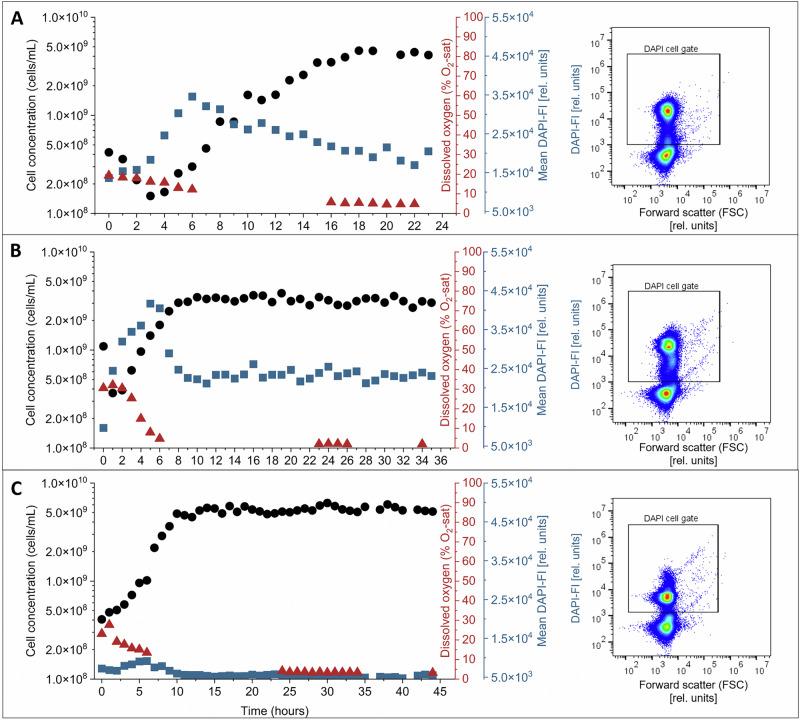
Automatic on-line flow cytometric monitoring of cell growth in continuous bioreactors for three bacterial strains. Cell concentration (black) and dissolved oxygen concentration (red) were measured hourly at a dilution rate of *D* = 0.25 h^−1^, *T* = 30 °C, and 250 rpm. Mean DAPI-FI (blue) at each time point was calculated as the average DAPI-FI of all events within the cell gate. **A** *Bradyrhizobium* sp. **B** *E. coli* and (**C**) *S. rhizophila*. The right-hand 2D dot plots illustrate representative DAPI-stained cells versus forward scatter at 20 h, with the same cell gate used to derive both cell concentration and mean DAPI-FI.

The strains *Bradyrhizobium* sp., *E. coli* and *S. rhizophila* were cultivated in continuously operated bioreactors and monitored by automated on-line flow cytometry. The time-resolved experiments described in this section were performed at a dilution rate of *D* = 0.25 h^−1^ with hourly sampling (Fig. 1). At *D* = 0.25 h^−1^, the residence time is 1/D = 4 h, and in the balanced phase of the continuous reactor setup the specific growth rate equals the dilution rate (*µ* = *D* = 0.25 h^−1^). After inoculation and exponential growth, all strains showed an adaptation phase followed by a balanced phase. Operationally, stability was defined as at least three consecutive sampling points (hourly) without significant changes in cell concentration and the monitored cytometric readouts. During the adaptation phase, the instantaneous growth rate can deviate from D, and *µ* = D applies to the balanced phase. The transition into the balanced phase occurred after 18 h for *Bradyrhizobium* sp., after 8 h for *E. coli*, and after 10 h for *S. rhizophila*. By 20 h, corresponding to five residence times at *D* = 0.25 h^−1^, all three cultures were in the balanced phase.

During the adaptation phase at *D* = 0.25 h^−1^, the strain *Bradyrhizobium* sp. immediately started to grow and continued until 18 h when the culture transitioned into the balanced phase, stabilizing at 4.22 × 10^9^ ± 1.61 × 10^8^ cells/mL (Fig. 1A). Consistent with elevated DNA synthesis during early adaptation, the mean DAPI-FI per cell increased and reached a maximal value of 3.46 × 10^4^ [rel. units] at 6 h. Afterward, the mean DAPI-FI gradually decreased until it balanced at 1.85 × 10^4^ ± 1.81 × 10^3^ [rel. units] between 18 and 24 h. The high growth activities were accompanied by a decrease in oxygen concentration to 5.1 ± 0.4% O₂-sat in the balanced phase.

The strain *E. coli* showed a different behavior at *D* = 0.25 h^−1^ (Fig. 1B). Although this bioreactor was also inoculated to the same OD of 0.05, growth started faster and the culture reached the balanced phase after 8 h, stabilizing at 3.14 × 10^9^ ± 2.39 × 10^8^ cells/mL. The high proliferation and uncoupled DNA synthesis during the first hours are demonstrated by the massive increase in mean DAPI-FI to 4.17 × 10^4^ [rel. units] during the adaptation phase until it levels to clearly lower values in the balanced phase (2.32 × 10^4^ ± 1.12 × 10^3^ [rel. units]). This adaptation period was accompanied by a marked decrease in dissolved oxygen concentration to nearly zero, consistent with high respiratory and metabolic activity during rapid proliferation.

The strain *S. rhizophila* showed a similar fast adaptation to bioreactor conditions at *D* = 0.25 h^−1^ (Fig. 1C). Starting also with an of OD_700 nm = 0.5 cm_ = 0.05, adaptation to bioreactor conditions was rapid and quickly transitioned into exponential growth until it reached the balanced phase after 10 h, at a cell concentration of 5.38 × 10^9^ ± 3.66 × 10^8^ cells/mL. The nearly doubled cell number compared to the *E. coli* strain was expected because the individual cell size of *S. rhizophila* is much smaller. Therefore, the bioreactor was inoculated with a higher initial cell number^34^. The mean DAPI-FI values were significantly lower than those of the other strains at 5.17 × 10^3^ ± 3.37 × 10^2^ [rel. units] due to the lower DNA content per cell, but peaked similarly during the adaptation phase (9.13 × 10^3^ [rel. units]). A decrease in dissolved oxygen levels was observed, corresponding to the increased metabolic activity during cell growth.

**Fig. 2:**
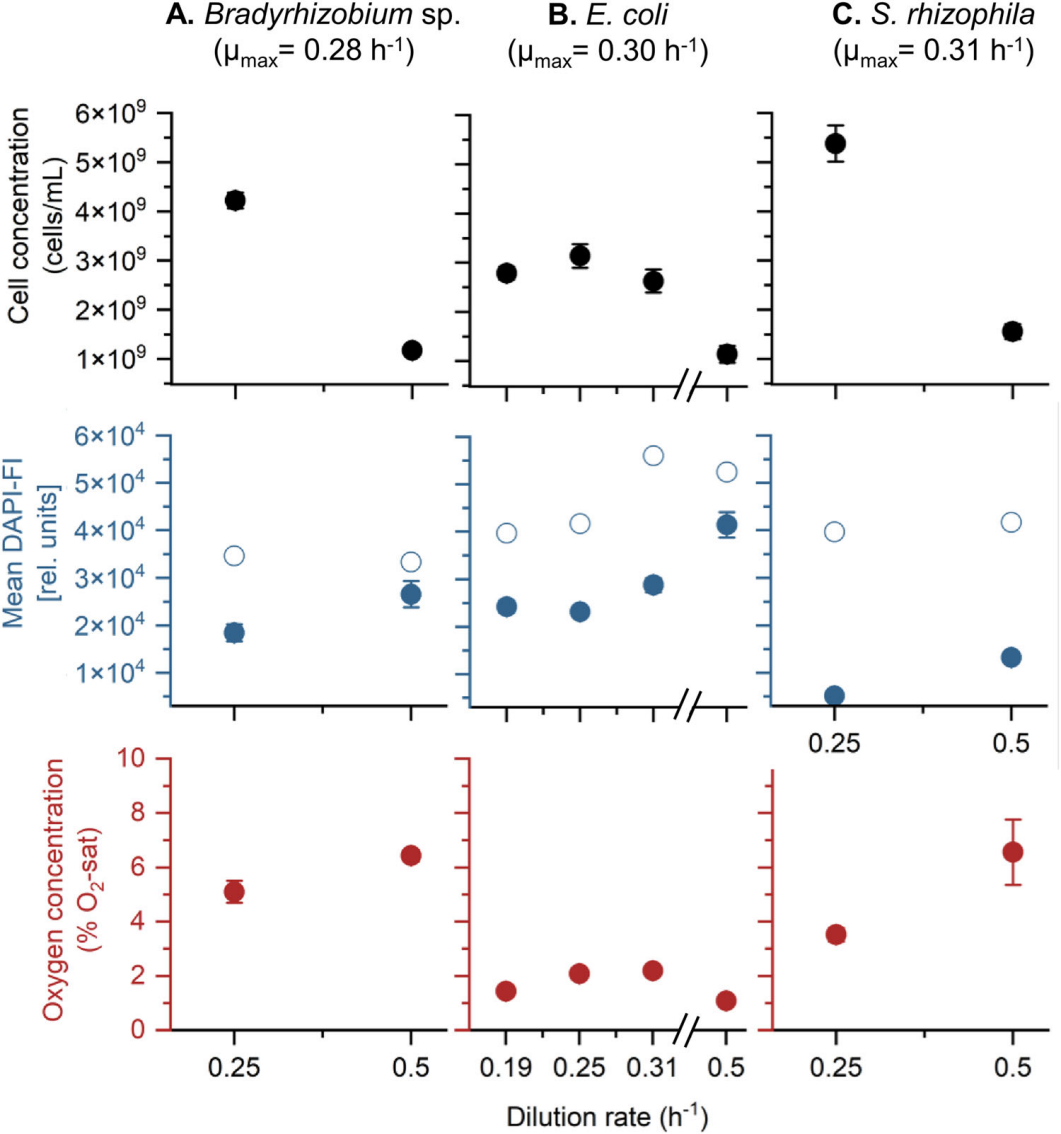
Growth behavior of three bacterial strains at different dilution rates. The values of cell concentration (black), mean DAPI-FI (blue), and oxygen concentration (red) were calculated as the mean values and standard deviation (SD) during the balanced phase of cultivation. Peak DAPI-FI (open blue) was determined as the maximum DAPI-FI reached over the entire experiment. **A** *Bradyrhizobium* sp., **B** *E. coli*, **C** *S. rhizophila*. Time-resolved data for the individual experiments are shown in Fig. 1 and SI 1–3. µmax was determined in batch cultivations in LB medium using 1 mL cultures grown in a 24-well plate.

In order to prove the sensitivity of the on-line cytometric analysis procedure, the three strains were also cultivated at the higher dilution rate of *D* = 0.5 h^−1^. *E. coli* was additionally grown at *D* = 0.19 h^−1^ and 0.31 h^−1^. These dilution rates correspond to residence times of 2.0 h (*D* = 0.50 h^−1^), 3.23 h (*D* = 0.31 h^−1^), and 5.26 h (*D* = 0.19 h^−1^), and in the balanced phase the corresponding growth rates are *µ* = *D*. The dilution rate of *D* = 0.5 h^−1^ was higher than the estimated *µ*_max_ values for each strain, which were determined to be 0.28 ± 0.02 h^−1^ for *Bradyrhizobium* sp., 0.3 ± 0.01 h^−1^ for *E. coli*, and 0.31 ± 0.03 h^−1^ for *S. rhizophila* in batch cultivations of the same medium. Therefore, a washout of the three strains was expected. Instead, cells were still detected (although at a lower concentration) in the balanced phase accompanied by even higher DAPI-FI values (Fig. 2 and SI 1). At *D* = 0.5 h^−1^, all three strains showed relatively short adaptation phases, earlier transitions into balanced growth, but substantially lower steady-state cell concentrations compared with *D* = 0.25 h^−1^.

*Bradyrhizobium* sp. initiated adaptive growth after 4 h and reached equilibrium within 14 h, stabilizing at 1.18 × 10^9^ ± 1.15 × 10^8^ cells/mL. Mean DAPI-FI rose rapidly, peaking at 4 h (3.33 × 10^4^ [rel. units]) and stabilizing at 2.66 × 10^4^ ± 2.76 × 10^3^ [rel. units], accompanied by still 6.43 ± 0.21% O_2_-sat levels (Fig. 2, SI 1A). *E. coli* showed a 10 h of adaptive growth phase, reaching only one-third of the biomass observed at *D* = 0.25 h^−1^ (1.14 × 10^9^ ± 1.69 × 10^8^ cells/mL). Mean DAPI-FI peaked higher than before (5.26 × 10^4^ [rel. units] at 9 h) and stabilized at roughly double the fluorescence seen at the lower dilution rate (4.15 × 10^4^ ± 2.67 × 10^3^ [rel. units]). Dissolved oxygen dropped to near-zero saturation levels (Fig. 2, SI 1B).

*S. rhizophila* exhibited a longer adaptation phase (10 h) and 8 h of exponential growth, reaching only one-third of the cell concentration measured at *D* = 0.25 h^−1^ (1.56 × 10^9^ ± 1.51 × 10^8^ cells/mL). Mean DAPI-FI increased continuously, peaking at 9 h (1.87 × 10^4^ [rel. units]) and stabilizing at 1.33 × 10^4^ ± 8.30 × 10^2^ [rel. units], corresponding to a 2.5-fold increase over *D* = 0.25 h^−1^ (Fig. SI 1C). *S. rhizophila* did not fully consume the supplied oxygen, maintaining 6.56 ± 1.20% O_2_-sat levels (Fig. 2 and S1C).

For *E. coli*, the same experiment was also conducted with an intermediate dilution rate of *D* = 0.31 h^−1^ which is near the *µ*_max_ value and a lower dilution rate of *D* = 0.19 h^−1^. Under *D* = 0.31 h^−1^ conditions, the cell concentrations were similar to those of *D* = 0.25 h^−1^ with only a slight decrease to 2.63 × 10^9^ ± 2.31 × 10^8^ cells/mL. The same was found for the mean DAPI-FI values with 2.88 × 10^4^ ± 1.50 × 10^3^ [rel. units] in the balanced phase (Fig. 2, SI 2). In addition, the oxygen consumption was in the same range. At *D* = 0.19 h^−1^, the cell concentration during the balanced phase (2.79 × 10^9^ ± 1.34 × 10^8^) was comparable to that observed at *D* = 0.31 h^−1^, and slightly lower than at *D* = 0.25 h^−1^. The DAPI-FI value (2.43 × 10^4^ ± 1.34 × 10^3^ [rel. units]) and the oxygen concentration remained within the same range as those recorded at *D* = 0.25 h^−1^ and 0.31 h^−1^ (Fig. 2, SI 3). Taken together, these data show that the online cytometric workflow can reproducibly resolve dilution-rate-dependent shifts in culture state, reflected in changes in mean DAPI-FI (DNA-content related) and cell concentration, and can clearly differentiate dilution-rate-dependent growth characteristics across conditions.

### Online cytometry as a sensor for bacterial growth by the analysis of the replication activity percentage

**Fig. 3:**
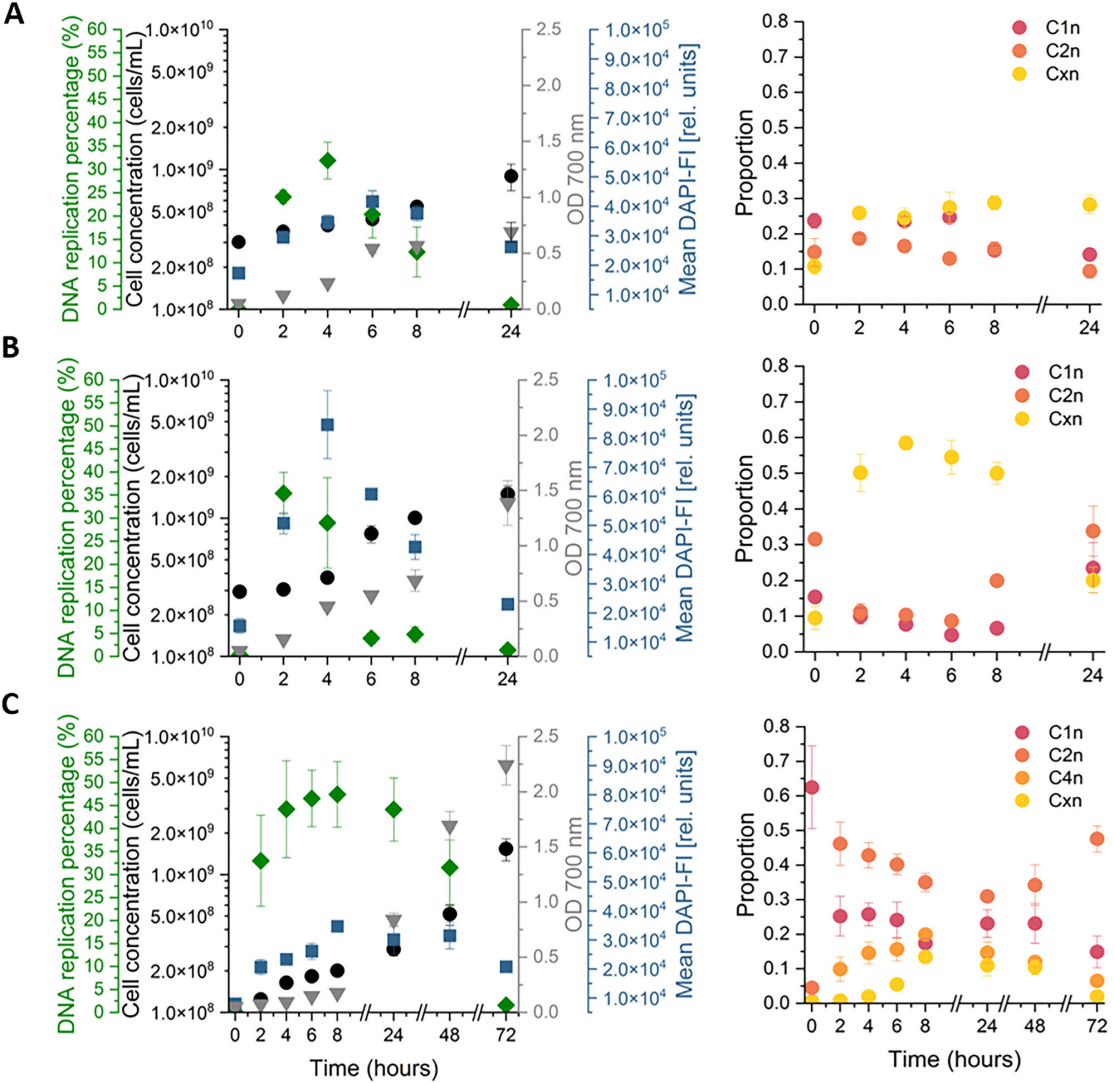
Automatic flow cytometric analysis of cell concentrations and dynamics of individual cell parameters (left) and subpopulation (right) of the three bacterial strains during batch cultivation. The graphs show the calculated mean values and the error bars representing the standard deviation of three independent biological experiments. Cells were cultivated in 24-well plates and sampled bihourly by the OC-300. For each strain, the left panels show DNA replication percentage (Alexa 488-EdU-positive cells, green), total cell concentration (cells/mL, black), OD700 (gray) and mean DAPI-FI (rel. units, blue). The right panels show the proportions subpopulations with different chromosome numbers over time: C1n, C2n, and Cxn for *Bradyrhizobium* sp. (**A**) and *E. coli* (**B**), and C1n, C2n, C4n, and Cxn for *S. rhizophila* (**C**). EdU was added at 7.5 µg/mL for *Bradyrhizobium* sp. and 3.75 µg/mL for *E. coli* and *S. rhizophila*.

In this study, we were particularly interested in following bacterial growth in bioreactor systems by the analysis of the DNA replication percentage. In addition to DAPI, which labels the number of chromosomes in each cell, we have used an Alexa 488-EdU derivative that specifically identifies cells in the process of replicating their DNA. To validate this method, we conducted in a first step a series of batch cultures, which were automatically sampled and measured. The cultures included individual strains of *Bradyrhizobium* sp., *E. coli*, and *S. rhizophila*, respectively, grown in 24-well plates at 30 °C and 150 rpm. Additionally, 100 µL samples were taken bihourly to measure OD at 700 nm. The results are shown in Fig. 3.

The automated procedure successfully performed the double staining procedure with DAPI and Alexa 488-EdU, enabling calculation of the DNA replication percentage (Formula 1). For all three strains, the DNA replication percentage showed a sharp and immediate increase directly after inoculation, reaching the peaks at 2 h and 4 h, respectively. This trend suggests an initial phase of rapid cellular replication activity followed by peak mean DAPI-FI postponed by a few hours. A slowdown in DNA synthesis activity was observed over the following hours as the bacterial cells entered the stationary phase. Consistent with these data, the cell concentrations showed a slow but steady increase throughout the observation period, starting at 3.03 × 10^8^ ± 8.41 × 10^6^ cells/mL at 0 h and rising to 8.98 × 10^8^ ± 1.92 × 10^7^ cells/mL at 24 h for *Bradyrhizobium* sp. and starting at 2.94 × 10^8^ ± 2.08 × 10^7^ and rising to 1.49 × 10^9^ ± 2.23 × 10^8^ cells/mL for *E. coli* (Fig. 3AB). In the case of *S. rhizophila* (Fig. 3C), the DNA replication percentage remained high and unchanged up to 48 h after the fast increase in the first 4 h. Only after 72 h the DNA replication activity dropped to nearly zero. Interestingly, once replication ceases at 72 h, a sharp increase in cell concentration was observed. This suggests increased cell division activity at the end of the log-phase of growth, resulting in a sudden rise in cell number to 1.53 × 10^9^ ± 2.74 × 10^8^ cells/mL. This correlation between DNA replication and cell concentration highlights the effectiveness of the automated procedure in capturing dynamic bacterial growth patterns over time.

Additionally, the proportions of cells with different chromosome numbers were distinguished by DAPI staining, adding another dimension to the analysis. The C1n, C2n, and Cxn subpopulations in *Bradyrhizobium* sp. and in *E. coli*, as well as the C1n, C2n, C4n, and Cxn subpopulations in *S. rhizophila*, were separated by applying the DAPI-FI gates (Fig. SI 4). The dynamics of these subpopulations were followed over the course of batch cultivation (Fig. 3, right panels). In *Bradyrhizobium* sp. the proportions of the subpopulations remained comparatively stable, indicating a more homogeneous distribution of cell chromosome contents over time (Fig. 3A). In contrast, *E. coli* showed pronounced shifts, with an increase of higher DAPI-FI subpopulations (Cxn) during the period of active growth, consistent with intensified DNA replication percentage (Fig. 3B). During prolonged cultivation, cells of *S. rhizophila* gradually shifted from the low-DNA quantity C1n subpopulation to the higher-chromosome number subpopulations (C2n, C4n, and Cxn), suggesting a continuous but slower buildup of cells with multiple chromosome equivalents (Fig. 3C). In both *E. coli* and *S. rhizophila*, the Cxn subpopulation with the highest DNA content increased during phases of high replication activity and declined afterwards, closely matching the dynamics of the Alexa 488-EdU signal, thereby demonstrating that the Alexa 488-EdU stain provides an accurate readout of the DNA replication state of the cells.

The next step was to integrate this procedure into a continuous bioreactor setup. We chose the *D* = 0.25 h^−1^ again to follow growth below *µ*_max_ values to avoid the washout points and to guarantee balanced growth conditions. As before, cell concentration, mean DAPI-FI, and DNA replication percentage were determined automatically using the controlled continuous bioreactor system. EdU was added to the culture media at a concentration of 3.75 µg/mL for all three strains. The oxygen concentration was measured as a standard. The automated flow cytometry procedure then sampled and performed the double staining of the bacterial cells every hour as before.

**Fig. 4:**
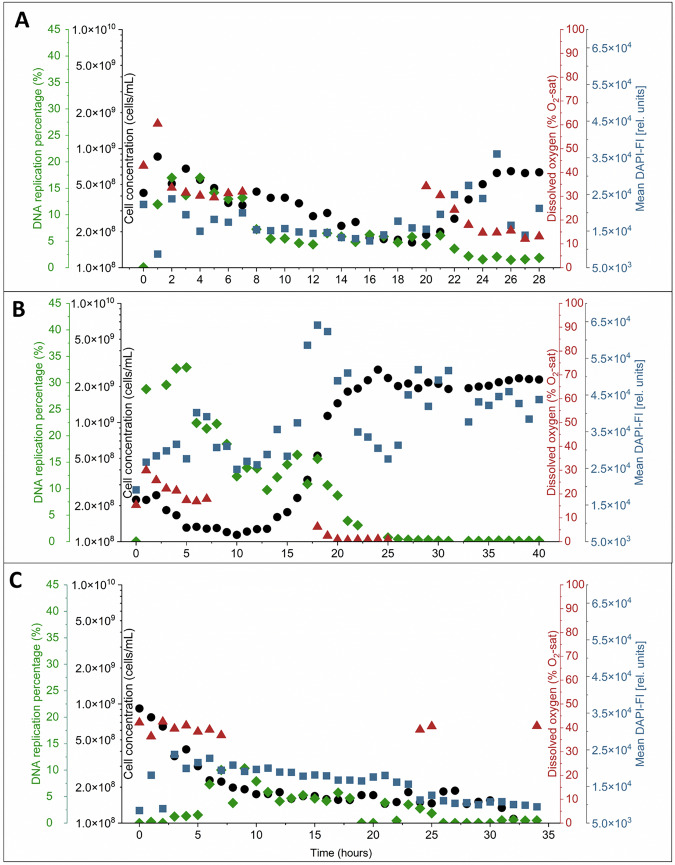
Automatic online flow cytometric monitoring of cell growth in continuous bioreactors for three bacterial strains. Cell concentration (black), DNA replication percentage (green) and dissolved oxygen concentration (red) were measured hourly at a dilution rate of *D* = 0.25 h^−1^, *T* = 30 °C, and 250 rpm. Mean DAPI-FI (blue) at each time point was calculated as the average DAPI-FI of all events within the cell gate. **A** *Bradyrhizobium* sp. **B** *E. coli* and **C** *S. rhizophila*.

In the experiment with *Bradyrhizobium* sp. (Fig. 4A), we observed that the cell concentration remained low but stable in the initial hours, without a significant increase. It remained low until the 20-h mark, when finally entered a short growth phase of 5 h. During this period, the cell concentration recovered and reached the initial inoculation value of 6.31 × 10^8^ ± 1.07 × 10^7^ cells/mL which is nearly 6.7-fold lower compared to the same experiment without the EdU application (Fig. 1A). Oxygen consumption followed the same pattern. No oxygen was consumed during the decrease in cell number, but consumption slightly increased along with the slow growth after 20 h. The DAPI-FI values supported these data. However, the DNA replication percentage showed a different behavior. While it was relatively high during the first 7 h at about 15%, it decreased to about 5% and after 20 h to even 2%, where it stabilized for the rest of the experiment. This indicated a very low proliferation activity.

For *E. coli* (Fig. 4B), we observed a different behavior. The DNA replication percentage reached the highest value within the first 5 h of the experiment (33%), then decreased slowly but continuously over the following hours, dropping to around 5% at 20 h. During the first 20 h, no significant increase in cell concentration was observed although the DAPI-FI increased sharply starting at 15 h from 3.3 × 10^4^ [rel. units] to a peak value at 17 h with 6.40 × 10^4^ [rel. units]. Only at the 20-h mark, the massive exponential growth phase became apparent by cell number values, lasting for 5 h, during which the cell concentration increased rapidly, reaching the balanced phase with 2.17 × 10^9^ ± 2.16 × 10^8^ cells/mL at 25 h. The balanced phase was accompanied by a significant decrease in DNA replication percentage, stabilizing only at around 0.5%. This behavior was similar to *Bradyrhizobium* sp. (Fig. 4A), where a period of higher DNA replication activity was followed by a decrease. Different from the *Bradyrhizobium* sp., the oxygen concentration was nearly zero, pointing still to active metabolism.

In the case of *S. rhizophila* (Fig. 4C), both cell concentration and DNA replication activity slowly reached the washout point. No oxygen was consumed. After a brief increase in cell number during the first 5 h, accompanied by a similar brief increase in DNA replication activity, all growth seemed to slow down. Both abiotic and cellular parameters were not in equilibrium.

**Fig. 5:**
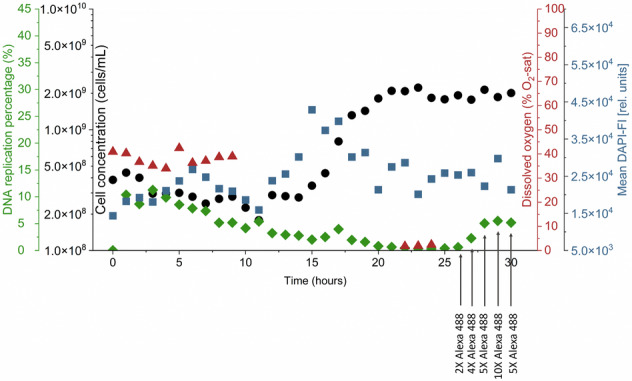
Automatic online flow cytometric monitoring of cell growth in continuous bioreactors for *E. coli.* Cell concentration (black), DNA replication percentage (green) and dissolved oxygen concentration (red) were measured hourly at a dilution rate of *D* = 0.25 h^−1^, *T* = 30 °C, and 250 rpm. Mean DAPI-FI (blue) at each time point was calculated as the average DAPI-FI of all events within the cell gate. Time points from 26 until 30 h indicate the different concentrations of Alexa 488 dye used in the Alexa 488 reaction mix in comparison with the original mix (Table 1).

The DNA replication percentage was reliably measured under batch cultivation conditions. However, this approach was not feasible for the tested species under continuous cultivation conditions. To exclude that the reason for low Alexa 488-EdU signals was a limited concentration of the Alexa dye we added 2-fold to 10-fold concentrations of the dye in the supposed balanced phase of growth. In the case of *E. coli*, the percentage of detectable DNA replication percentage was restricted to the initial hours of the experiment. During the later stages, when cell concentration was at its highest, a tenfold increase in dye concentration was required to resolve Alexa 488-EdU positive cells (Fig. 5). For *Bradyrhizobium* sp., the DNA replication percentage was monitored, but the presence of EdU seemed to negatively impact its growth. As a result, only a short growth phase was observed, with low cell concentrations throughout. Nearly no DNA replication activity was observed for *S. rhizophila*.

## Discussion

Automated online flow cytometry allows for the real-time monitoring of population and microbial community dynamics in various natural and managed settings. Real-time, single-cell-level indicators are especially interesting in biotechnological setups, as they can support control strategies in various types and scales of reactor systems. The most important information is the cell growth because either the biomass itself may be the product, or it may serve as a biocatalyst to support the synthesis of a desired product. Because many industrial bioprocesses are product-oriented, growth-related readouts should primarily be interpreted as indicators of biomass and culture state. Depending on the process, they may correlate with productivity, for example, in growth-associated production or under strong production burdens, but they are not a universal proxy for product formation, which remains product and process-specific and typically requires dedicated analytics. Strong information on biomass is already provided by the cell count, as it indicates the number of biocatalyst cells in a reactor environment. The activity of the biocatalyst is identified in this study by fluorescent labels that highlight the growth states of cells and the number of cells that are actively replicating. Therefore, the combination of these two dyes pinpoints cells that have paused in the cell cycle and those that are actively replicating their DNA. In bioreactors, changes in cell concentration inform whether the applied dilution rate maintains the population within the desired biomass range, while persistently low mean DAPI-FI may suggest nutrient limitation, indicating the need for stronger or more specific feeding strategies. Conversely, a decrease in the DNA replication percentage may signify inhibitory cell replication phenomena. Such feedback based on single-cell cytometric readouts complements conventional process parameters and can improve process stability and product quality^37^. It should be noted that traditional bulk biomass parameters, such as OD values, do not always correspond to varying cell numbers across strains and specific environments due to differences in size, morphology, and bioreactor setting^34,38–40^, which is another reason why automated flow cytometry monitoring is a superior option.

As outlined in the Introduction, several established monitoring strategies exist for continuous bioprocesses. Here, the key added value of automated online flow cytometry is that it combines a direct biomass metric (cell concentration) with single-cell physiological information in the same measurement, rather than relying on population-averaged proxies alone. This enables detection of shifts in culture state, for example, changes in DNA-content related DAPI metrics that can precede visible changes in total cell concentration, and thereby supports earlier intervention in continuous operation. The approach therefore, complements conventional process variables, but it also comes with practical trade-offs, including instrument and method complexity, reagent use, and the fact that product formation remains product-specific and requires dedicated analytics^34,37,38^.

Our automated configuration, which includes sampling, fixation, permeabilization, staining, and transportation to the flow cytometer, provides shorter processing times and is more user-independent compared to manual flow cytometry procedures^34^. We record cell counts together with per-cell DAPI fluorescence, and use the mean DAPI-FI as a proxy for DNA content per cell. In the case of the continuous bioreactor experiments, the rapid online fixation, permeabilization, staining, and dilution sequence prioritized robustness and temporal resolution, but discrete chromosome-equivalent subpopulations could not be consistently resolved in the DAPI distributions; therefore, we focused on total cell counts and mean DAPI-FI as the most reliable readouts. The two parameters were shown to serve effectively as control variables for cell growth in multiple bioreactor systems and provided more specific information than bulk parameters, such as OD and dissolved oxygen, which offer a more general readout of overall culture conditions^38,41^. Accordingly, the very low dissolved oxygen values observed in this study most likely reflect oxygen transfer limitations in the 10-mL reactor under air aeration without active DO control, rather than a general characteristic of continuous cultivation systems. The mean DAPI-FI monitored cell growth hours earlier than OD or dissolved oxygen values. The rise in DAPI-FI, as is shown for all three strains in Figs. 1 and 3, as well as in Figs. SI 5–7, is a strong indicator that a population underwent cell-cycle progression. The signal precedes even the increase in cell number, providing an opportunity for timely intervention and decision-making. These interpretations align with validated cytometric practice: DNA-binding dyes yield DNA-content histograms that track genome copy number and cell-cycle phase^24,32^.

Further insight into cell cycle dynamics is required when continuous bioreactor systems are operated and controlled by automated flow cytometry. During balanced growth phases, the mean DAPI-FI values remain unchanged and do not indicate the proportion of cells undergoing active replication. The Alexa Fluor 488-EdU signal was therefore identified as a potential method of identifying and quantifying cells actively replicating DNA at the time of sampling.

In batch cultivations, the EdU-positive fraction peaked before the mean DAPI-FI values in all three strains (Fig. 3A), verifying the assumption that this signal is more specific and able to determine the DNA replication percentages to highlight the most active cells in a population. In *E. coli* and *S. rhizophila*, the fraction of the Cxn subpopulation, which has the highest DAPI-FI and thus the highest DNA content, increased after the Alexa 488-EdU signal and then declined once the replication burst decreased. This pattern is consistent with cells first accumulating multiple chromosome equivalents during intense DNA replication and subsequently redistributing into lower-DAPI subpopulations (C1n/C2n/C4n) as cell division proceeds. Hence, the dynamics of Cxn closely mirror the EdU-based replication readout, supporting that the Alexa 488-EdU signal provides an accurate and sensitive measure of the DNA replication state at the population level. *Bradyrhizobium* sp. also showed an initial increase in the frequency of the Cxn subpopulation, along with the Alexa 488-EdU signal. However, unlike the other two strains, the Cxn subpopulation remained at a high level, which was not reflected by the decreasing EdU-based replication readout. *Bradyrhizobium* strains form multicellular aggregates, or flocs, in liquid culture through exopolysaccharide-mediated autoaggregation^42–44^, which explains the apparent high DNA Cxn fraction despite the absence of replication. This highlights the advantage of the Alexa 488-EdU approach, which distinguishes between replication activity from paused states in the cell cycle.

However, the analysis of the DNA replication percentage was mostly unsuccessful for the continuous bioreactor experiments. While *E. coli* exhibited a recognizable response to the Alexa 488-EdU dye (Fig. 4B), the other two strains did not. A continuous growth of *S. rhizophila* (Fig. 4C) was most severely inhibited, preventing sufficient growth to reach equilibrium and resulting in washout. The growth of *Bradyrhizobium* sp. was also hampered. Even *E. coli* showed delayed growth in both DAPI-FI and cell number, as well as a long adaptation period, compared to the same experiment performed without EdU (Fig. 1B). For *E. coli* only, the DNA replication percentage could be measured up to 22 h, while the mean DAPI-FI and cell numbers stabilized at values slightly below those of the control experiment (Figs. 1B and 4B) where the balanced growth state was established already after 10 h (Fig. 1B). This behavior suggested that the Alexa concentration was too low to label the EdU in the DNA of replicating cells. However, increasing the Alexa 488 concentration by up to 10-fold did not result in a similar increase in DNA replication percentage to that initially observed (5.1%) or expected from the batch culture (35.4%, Figs. 3B and 5). Therefore, a lack of the Alexa 488 dye was not the reason for the low EdU readouts. Nevertheless, as with the data obtained for batch cultures, the Alexa 488-EdU signal was detected earlier than the DAPI-FI signal and the increase in cell counts in continuous cultivations of *E. coli* when the automated online flow cytometry was used.

The Alexa 488-EdU staining procedure was originally developed to mark DNA replication in human cell lines^27,45^. To adapt the method to bacteria, we tested ten Gram-positive and Gram-negative strains using the online staining procedure (see List SI 1), but with limited success. Only *Bradyrhizobium* sp., *E. coli*, and *S. rhizophila* showed Alexa 488-EdU fluorescence. We demonstrated that *E. coli* was particularly amenable to the method when the cells were fixed using a standard protocol that included centrifugation steps (see Methods, Paraformaldehyde (PFA)/ethanol (EtOH) fixation and DAPI staining for chromosome number analysis, Fig. SI 8). It has been reported that EdU incorporation inhibits the growth of both human and bacterial cells^46,47^. The presence of EdU appears to significantly affect bacteria growth in continuous bioreactors, where EdU is supplied continuously, as opposed to batch cultures, where EdU is only added at the beginning. Therefore, we do not recommend using Alexa 488-EdU staining to determine the DNA replication percentage in bacterial bioprocess monitoring due to the limited applicability of the Click-iT reaction to multiple strains, its growth-inhibiting effects, and its high cost. However, the procedure demonstrated that a double-staining approach can effectively be established to characterize bacterial physiological states using automated online flow cytometry connected to the OC-300 device.

There are further insights to be gained from automated online flow cytometry in relation to dilution rate and washout behavior. In bioprocesses, understanding the relationship between dilution rate (D) and cell concentration is crucial for optimizing reactor conditions^1,39^. Our experiments revealed that cell concentration during the balanced phase was higher at lower dilution rates (*D* = 0.19 h^−1^, 0.25 h^−1^ and 0.31 h^−1^) than at the higher dilution rate (*D* = 0.5 h^−1^). This indicates that at D = 0.5 h^−1^ the system approaches or exceeds the maximum specific growth rate (*µ*_max_) of the strains, leading to a reduced but still stable cell concentration during the balanced phase (Fig. 2). Balanced (steady-state) growth in a continuous reactor setup implies that the specific growth rate equals the dilution rate (*µ* = *D*), and it should not be interpreted as growth at µmax, which is an upper bound measured under non-limiting conditions. Despite the fact that the dilution rate was above *µ*_max_ (as determined in batch culture), no washout of the cells was observed, and high DAPI-FI indicated intense proliferation activity, which may have prevented complete washout.

From a theoretical standpoint, the dynamics of CSTRs (continuous stirred-tank reactor) predict washout when the dilution rate surpasses *µ*_max_, as indicated in classical models^48–50^. In practice, however, there is often a range of dilution rates beyond *µ*_max_ where washout does not occur but cell concentration is reduced until a critical dilution rate (*D*_c_) is reached; at *D*_c_, washout occurs and no further growth is possible^51–54^. Deviations from ideal CSTR behavior can be explained by several factors that alter microenvironments in bioreactors. Higher dilution rates can lead to nutrient imbalances or altered metabolic fluxes, and the components necessary for cell growth—such as carbon and nitrogen sources, oxygen, and cells—are never perfectly mixed. In our continuous reactor setups, increasing the dilution rate decreased the total cell concentration, but it also increased the mean DAPI-FI, indicating a higher DNA content per cell. Such behavior is expected for real CSTR experiments operated near D_c_, where higher dilution rates are associated with higher residual substrate concentrations and shorter residence times. Under these conditions, only the fastest-growing cells can keep up with the outflow; slower cells are washed out. Consequently, the population at D above µ_max_ is smaller but enriched with fast-growing cells, which is reflected in the increased mean DAPI-FI despite lower total cell numbers^55–57^. Therefore, by integrating automated online flow cytometry into bioreactor monitoring provides comprehensive, real-time insights into population dynamics that would otherwise be difficult to interpret. Suboptimal growth rates can be detected early, allowing proactive interventions to maintain bioreactor performance.

This study demonstrates that automated online flow cytometry is a powerful tool for monitoring and controlling bioreactor systems in real time. The approach overcomes the limitations of traditional offline detection methods by providing live cell population data, such as cell density and cell cycle states. By integrating a double-staining strategy, we used DAPI-FI to follow growth state and the Click-iT Alexa 488–EdU reaction to quantify DNA replication percentages. The combination of these two dyes pinpoints cells that have paused in the cell cycle and those that are actively replicating their DNA. Therefore, a decrease in the DNA replication percentage may indicate the cessation of cell cycle activities or the inhibition of cell cycle replication.

Our findings highlight DAPI-FI as an early marker of microbial growth that responds before cell numbers increase, enabling timely interventions to optimize bioprocess conditions in both batch cultures and continuous reactors operated at different dilution rates. In addition, the same cytometric readouts could be used to qualify seed cultures prior to inoculation by identifying an inoculation window enriched in replication-active cells, indicated for example, by elevated mean DAPI-FI (higher average DNA content per cell). Discrepancies between theoretical CSTR models and real-world bioreactor behavior have been shown, particularly in relation to dilution rate effects and washout dynamics. The second cell label, the Click-iT Alexa-488-EdU reaction, reliably detects fractions of actively replicating cells in batch cultures, even before the DAPI-FI signal. It can also differentiate between fractions of stalled and replicating cells in the cell cycle. However, under continuous cultivation conditions in which EdU is a component of the medium, the signal was only partially effective for *E. coli* and ineffective for other bacterial strains.

Nonetheless, the organization of the automated procedures—including sampling, dilution, permeabilization, fixation, double staining, and transportation of a sample to the flow cytometer—was demonstrated to be feasible with the OC-300. This renders the procedure applicable to other dye combinations. Ultimately, the future of running and optimizing industrial biotechnological processes is the strategic implementation of automated online flow cytometry to operate and control bioreactor systems in real time.

## Methods

### Cultivation of bacterial cells

The strains *Bradyrhizobium* sp. Leaf396-mScar, obtained from Schlechter et al.^58^, *Escherichia coli* K12 LE392 DSM 4230, and *Stenotrophomonas rhizophila* DSM 14405 (both obtained from the German Collection of Microorganisms and Cell Cultures (DSMZ), Leibniz Institute, Braunschweig, Germany) were initially cultivated on LB agar plates (Lysogeny Broth, Chemsolute, Renningen, Germany) at 30 °C for 72 h, starting from glycerol stock suspensions. Subsequently, 20 mL of liquid LB medium was inoculated with a single colony of each strain and incubated at 30 °C with shaking at 250 rpm for 24 h. After incubation, the optical density (OD_700 nm = 0.5 cm_) of the preculture was measured using an Ultraspec 1100 Pro spectrophotometer (Amersham Biosciences, Amersham, UK). The required volume to inoculate the main culture, whether for batch cultivation in a 24-well plate or for continuous culture in a bioreactor, was then calculated to achieve an initial OD_700 nm = 0.5 cm _= 0.05.

### Cultivation of bacterial cells in batch experiments

All strains were cultivated on LB medium. For experiments in which Alexa 488 fluorescence was to be measured, 5-ethynyl-2′-deoxyuridine (EdU) was added to the LB medium at an initial concentration of either 3.75 or 7.5 µg/mL. The LB medium was then inoculated with a sufficient amount of the preculture to achieve an initial OD_700 nm = 0.5 cm _= 0.05. The culture was transferred to a 24-well plate. Each well contained 1 mL of the inoculated medium. The plate was then incubated at 30 °C with shaking at 150 rpm for 24 h for both *E. coli* and *Bradyrhizobium* sp., and 72 h for *S. rhizophila*. To ensure sterility while allowing air exchange, the plate was covered with a “Breathe-easy” anti-evaporation foil (Merck KGaA, Darmstadt, Germany) and placed in an Incubator Hood TH 30 (Edmund Bühler GmbH, Bodelshausen, Germany). Growth was measured by initial OD_700 nm = 0.5 cm_ = 0.05 hourly up to 8 h, and then at 24 h, 48 h and 72 h.

### Cultivation of bacterial cells in a continuous bioreactor

Either LB medium or EdU-LB medium with initial concentrations of either 3.75 or 7.5 µg/mL EdU was prepared. Due to the high cost of the EdU chemical, a small bioreactor with a volume of 12 mL and a working volume of 10 mL was chosen. The respective medium was inoculated with an appropriate volume of preculture to reach OD_700__ nm = 0.5 cm_ = 0.05. The continuous bioreactor was maintained with a constant working volume of 10 mL at 30 °C and 250 rpm (Cimarec Poly 15 und Multipoint Stirrer, Thermo Fisher Scientific Inc., Waltham, MA, USA; Incubator Hood TH 120 25, Edmund Bühler GmbH, Bodelshausen, Germany). To maintain a constant volume, we ran one inflow (*F*_i_) of fresh medium and two outflows by two peristaltic pumps (LabN1-II peristaltic pump, Drifton A/S, Denmark), namely the continuous effluent (*F*_o_) and the automated sampling withdrawal (*F*_s_), set so that *F*ᵢ ≈ *F*ₒ + *F*_s_, with *F*_s_ = 1.2 mL/h sample collection. No active level or weight control was used. Volume constancy was verified visually at the 10-mL graduation mark at startup and periodically throughout the operation. The effective dilution rate was therefore *D* = (*F*ₒ + *F*_s_)/*V* = *F*ᵢ/*V*. For *D* = 0.25 h^−1^, feeding rates were *F*ᵢ = 2.5 mL/h, *F*ₒ = 1.3 mL/h, *F*_s_ = 1.2 mL/h. For *D* = 0.50 h^−1^, feeding rates were *F*ᵢ = 5.0 mL/h, *F*ₒ = 3.8 mL/h, *F*_s_ = 1.2 mL/h. For *D* = 0.31 h^−1^ and *D* = 0.19 h^−1^, feeding rates were 3.125 and 1.9 mL/h and outflows were 1.925 and 0.7 mL/h, respectively, with *F*_s_ kept at 1.2 mL/h in all cases. We tested two dilution rates, *D* = 0.25 h^−1^ and *D* = 0.50 h^−1^, for all three strains. Additional conditions at *D* = 0.31 h^−1^ and *D* = 0.19 h^−1^ were run only for *E. coli*. The peristaltic tubing used was Tygon LMT-55, with an inner diameter of 1.295 mm (Saint-Gobain S.A., La Defense, France). With the operating conditions used, the 10-mL bioreactor had an estimated mixing time of approximately 7 s. Mixing time was determined using a dye tracer test and defined as the time from dye addition until the reactor volume appeared visually homogeneous and remained stable. Dissolved oxygen (DO) was monitored and expressed as percent saturation relative to pure-oxygen solubility at 30 °C using the O₂ Sensorspot SP-PSt6-YAU (PreSens Precision Sensing GmbH, Regensburg, Germany). The sensor spot was read out non-invasively by optical interrogation based on luminescence quenching, and after two-point calibration (zero and oxygen-saturated water), the expected accuracy is within a few percent saturation under stable temperature conditions.

Aeration was maintained by keeping a constant oxygen pressure at 40 psi, and a 0.2 µm filter was used for air exchange (Labsolute, Renningen, Germany). The DO was read out with the optical sensor spot during staffed hours because it was not feasible to continuously sense the DO in situ due to the small reactor working volume (10 mL) and geometry. The spot was calibrated against nitrogen-deoxygenated water (zero) and pure-oxygen–saturated water.

We defined a stable phase operationally as an interval during which process variables showed no appreciable trend under a constant dilution rate. This included flat time courses in OD and oxygen consumption as well as unchanging cytometric readouts. These intervals were identified directly from the time series and used qualitatively to assess signal stability across channels.

### Paraformaldehyde (PFA)/ethanol (EtOH) fixation and DAPI staining for chromosome number analysis

To perform an analysis of subpopulations with varying chromosome numbers of pure strains, DAPI staining was carried out following paraformaldehyde (PFA)/EtOH fixation, as described by Cichocki et al.^34^. In short, cells from the batch cultures were sampled hourly and adjusted to an OD_700 nm = 0.5 cm _= 0.5 in phosphate buffer (289 mM Na_2_HPO_4_ and 128 mM NaH_2_PO_4_ with double-distilled water, pH 7), then centrifuged at 3200 × *g* at 4 °C for 10 min. The cells were incubated for 30 min in 2 mL of a 2% PFA solution at room temperature (RT). After incubation, the PFA was removed by centrifugation (3200 × *g*, 4 °C, 10 min), and the cells were resuspended in 2 mL of a 70% EtOH fixation solution for at least 1 h at RT. The fixed cells were then washed with PBS buffer by centrifugation (3200 × *g*, 4 °C, 10 min) and adjusted to 2 mL with an OD_700 nm = 0.5 cm _= 0.04. The PBS was discarded by centrifugation (3200 × *g*, 4 °C, 10 min), and 2 mL of a 0.24 µM DAPI staining solution in PBS buffer, diluted from a DAPI stock solution (143 µM DAPI dissolved in 100 µL dimethylformamide and then in double-distilled water), was added and incubated overnight. After incubation, the sample was ready to be measured.

### DAPI and Alexa 488-EdU double staining procedure for automated determination of cell concentration, mean DAPI-fluorescence intensity (FI), and DNA replication activity analysis

Automated DAPI and Alexa 488-EdU (Thermo Fisher Scientific Inc., Waltham, MA, USA) staining were used to assess cell number, mean DAPI-FI, and DNA replication activity. This online process required fixation and permeabilization to ensure effective staining. The fixation method employed was a previously validated NaCl/NaN_3_/EtOH protocol^7^. The NaCl/NaN_3_/EtOH fixation was carried out by adding 475 µL of 30% NaCl, 35 µL of 20% NaN_3_ (both Merck KGaA, Darmstadt, Germany), and 100 µL of 70% EtOH (Chemsolute, Renningen, Germany) to 100 µL of the bacterial sample. This resulted in final concentrations of 20% NaCl, 1% NaN_3_, and 10% EtOH, with an incubation time of 10 min. Following fixation, a permeabilization step using Triton X (Merck KGaA, Darmstadt, Germany) was carried out. Specifically, 100 µL of the fixed cell sample from the previous step was treated with 200 µL of 0.5% Triton X-100 and incubated for 20 min. Subsequently, the double staining with DAPI and Alexa 488-EdU was carried out simultaneously. This entire procedure can be performed either manually by pipetting or automatically by the OC-300 automation unit (onCyt Microbiology, Zürich, Switzerland) without any necessary centrifugation step. Online DAPI staining was achieved using a 1 µM DAPI staining solution in PBS buffer. The Alexa 488 stain was performed using the “Click-iT EdU Cell Proliferation Kit for Imaging, Alexa Fluor™ 488 dye” (cn C10337 Thermo Fisher Scientific Inc., Waltham, MA, USA). From the kit the following amounts of chemicals were used to prepare one volume of reaction cocktail (Table 1).

**Table 1:** Click-iT reaction cocktail of Alexa Fluor-488 staining solution

Reagent	Volume (µL)
1x Click-iT EdU reaction buffer	34.4
Copper sulfate (100 mM)	1.6
Alexa Fluor 488 picolyl azide (10 mM)	0.096
Click-iT EdU buffer additive	4

Volumes for 1 sample.

Once the Alexa 488 staining solution had been prepared, the double staining procedure was performed. To do this, 200 µL of 1 µM DAPI staining solution and 40 µL of the Alexa 488-EdU reaction cocktail were added to 100 µL of the permeabilized cell suspension and incubated for 10 min. After, the stained cell suspension was diluted 1:20 in MilliQ water (IQ 7000 Ultrapure Lab Water System, Merck KGaA, Darmstadt, Germany) and measured immediately.

### Automated workflow for the online double staining of a bacterial culture

**Fig. 6:**
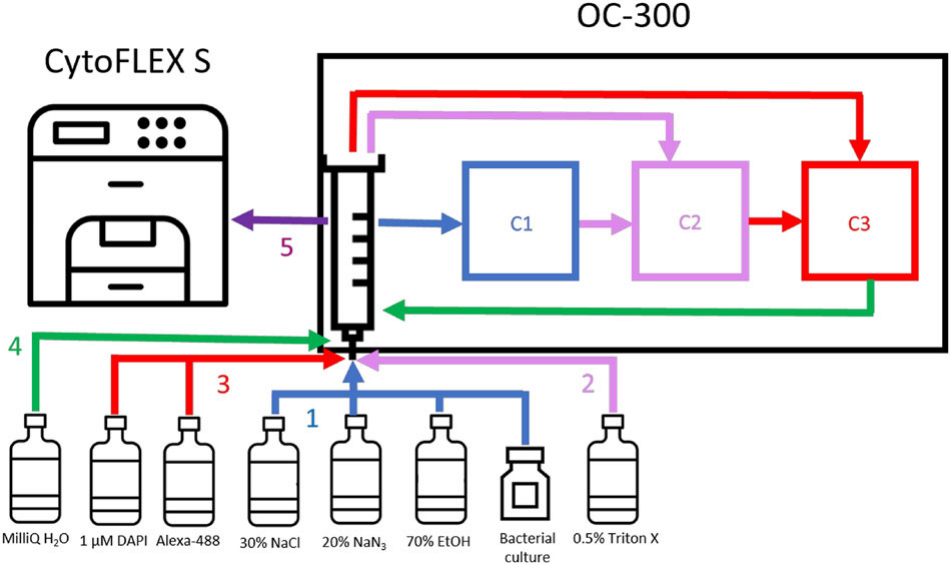
A schematic representing the workflow of automated sampling, fixation, permeabilization, double staining, dilution and measurement performed by the OC-300. (1) The sample drawn from the bacterial culture was moved to chamber 1 (C1) and fixed with NaCl (20%), NaN_3_ (1%), and EtOH (10%) for 10 min. (2) The fixed sample was moved from C1 to chamber 2 (C2) and permeabilized with 0.5% Triton X for 20 min. (3) After permeabilization, the sample was transferred from C2 to chamber 3 (C3) and stained with DAPI (1 µM) and Alexa 488-EdU reaction cocktail for 10 min. (4) The stained sample was diluted 1:20 with MilliQ water in the syringe. (5) Finally, the diluted sample was sent to the cytometer for measurement.

The double staining procedure outlined above was performed online by the OC-300 automation unit. For sampling, the OC-300 automation unit has a dedicated valve port connected by sterile tubing directly to either the batch culture or the continuous bioreactor, and then carried out the sequential steps of fixation, permeabilization, double staining, and final dilution. Once these steps were completed, the stained cell suspension was sent to the CytoFLEX S flow cytometer (Beckman Coulter, Brea, CA, USA) for measurement. After each measurement, the device cleaned itself and prepared for the next sample. Considering the fixation, permeabilization and staining times, and the cleaning process between samples, a new sample was drawn and measured every 60 min (Fig. 6).

### OC-300 automation unit

The OC-300 automation unit features two valves, each with twelve ports that allow for the intake and dilution of samples, as well as the addition of reagents via a syringe. These valves also link the incubation chambers within the device. Inside the three incubation chambers, bacterial samples can be mixed with various solutions to carry out required steps. The unit was controlled by the cyOn software (onCyt Microbiology, Zürich, Switzerland) and was connected to the bioreactor through a sampling port, while the flow cytometer was linked via the OC-300-CytoFLEX interface. In the case of 24-well plate experiments, the sampling tubing was inserted into the well at every sampling time and the sample was taken automatically by the OC-300 and processed.

### Cytometric analysis

The CytoFLEX S flow cytometer (Beckman Coulter, Brea, CA, USA), operated with the CytExpert software (Beckman Coulter, Brea, CA, USA), was equipped with 375 nm (60 mW), 488 nm (50 mW), and 638 nm (50 mW) lasers. The 488 nm laser was used to detect forward scatter (FSC) (488/8 nm band-pass), side scatter (SSC) (488/8 nm band-pass, trigger signal), and Alexa 488-EdU fluorescence (525/40 nm band-pass). The DAPI-FI (450/45 nm band-pass) was measured using the 375 nm laser for excitation. The fluidic system was operated at a constant speed of 60 µL/min. For optical calibration in the logarithmic range, 0.5 and 1.0 µm UV Fluoresbrite microspheres (Polysciences, Cat. Nos. 18339 and 17458, Warrington, PA, USA) and 0.5 and 1.0 µm Yellow Green Fluoresbrite microspheres (Polysciences, Cat. Nos. 17152-10 and 17154-10, Warrington, PA, USA) were used. Typical acquisition rates were a median of around 1000 events/s, with maximum rates up to about 5000 events/s depending on strain and time point.

### Bioinformatic tools

The cell concentration, relative subpopulation proportions and mean DNA-FI were calculated using the software FlowJo (BD Biosciences, Franklin Lakes, NJ, USA). This software defines gates that include cells with similar characteristics as subpopulations. FlowJo was also used to create the 2D dot plots. The creation of the barcode images for the analysis of different chromosome number subpopulations was done by the flowCyBar software^12^ embedded in the biTCa Analyze Tool graphical user interface (GUI) developed by Bruckmann et al.^59^. The time series graphs were made in the software Origin 2023 (OriginLab Corporation, Northampton, MA, USA).

### Calculation of DNA replication percentage

The online cytometric procedure combined the use of the two fluorescent dyes DAPI and Alexa 488-EdU to determine their respective proportions over time. We operationally defined DNA replication percentage as the fraction of DAPI-gated events that are Alexa 488-EdU-positive during the sampling interval:

$$DNA\,replication\,percentage=\,\frac{number\,of\,Alexa\,488-EdU\,positive\,events}{number\,of\,DAPI\,positive\,events}$$

**Formula 1**. Calculation of DNA replication percentage.

## Supplementary information

Supplementary Information

## Data Availability

All data presented in this work are available in the Zenodo repository, accessible in the following link: https://zenodo.org/records/16418399.
