# Supplementary material for: A double-staining automated flow cytometry method for real-time monitoring of bacteria in continuous bioreactors
Source: NPJ Syst Biol Appl. 2026 Apr 1;12:47. doi: 10.1038/s41540-026-00694-3 (PMC13046813; doi:10.1038/s41540-026-00694-3)
Supplement: Supplementary file 1 — Supplementary Information [file 41540_2026_694_MOESM1_ESM.pdf]

A double-staining automated flow cytometry method for real-time monitoring of bacteria in continuous bioreactors

Juan López-Gálvez[1], Erik Schönfelder[1], Hanna Mayer[1], Konstanze Schiessl[2], Marisa O. D. Silva[2], Hauke Harms[1] & Susann Müller[1,*]

[1] Department of Applied Microbial Ecology, Helmholtz-Centre for Environmental Research – UFZ, Permoserstr. 15, 04318 Leipzig, Germany

[2] onCyt Microbiology AG, Libernstrasse 24, 8112 Otelfingen, Switzerland

*Corresponding author: susann.mueller@ufz.de

Supplementary Information

List of Contents

Figure SI 1. Automatic on-line flow cytometric monitoring of cell growth in continuous bioreactors for three bacterial strains with D = 0.5 h^{-1} 2

Figure SI 2. Automatic on-line flow cytometric monitoring of cell growth in continuous bioreactors for *E. coli* with D = 0.31 h^{-1} 3

Figure SI 3. Automatic on-line flow cytometric monitoring of cell growth in continuous bioreactors for *E. coli* with D = 0.19 h^{-1} 3

Figure SI 4. Subpopulation gating of DAPI-FI [rel. units] versus forward scatter (FSC) [rel. units] in batch-cultivated strains.......... 4

Figure SI 5. Barcode plot illustrating the distribution of *Bradyrhizobium* sp. subpopulations with varying chromosome numbers during batch cultivation 5

Figure SI 6. Barcode plot illustrating the distribution of *E. coli* subpopulations with varying chromosome numbers during batch cultivation.......... 6

Figure SI 7. Barcode plot illustrating the distribution of *S. rhizophila* subpopulations with varying chromosome numbers during batch cultivation 7

Figure SI 8. 2D flow cytometric plots of an *E. coli* culture sampled at various time points. The cells were batch-cultivated in a 24-well plate, manually sampled, and processed.......... 8

List SI 1. List of bacterial strains tested for the Alexa 488 and DAPI double staining procedure.......... 8

References 8

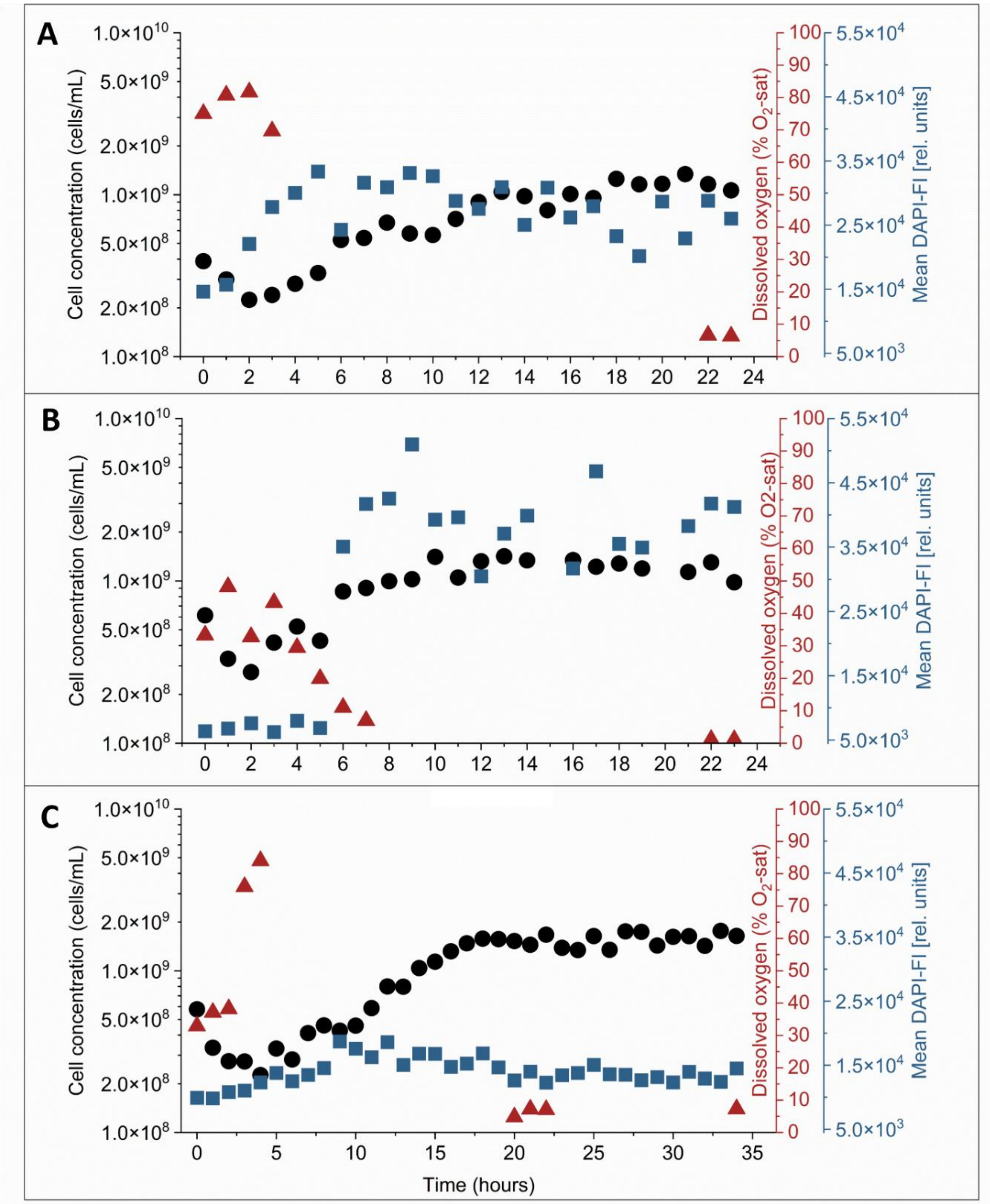

Figure SI 1. Automatic on-line flow cytometric monitoring of cell growth in continuous bioreactors for three bacterial strains with D = 0.5 h^{-1}. Cell concentration (black) and dissolved oxygen concentration (red) were measured hourly at a dilution rate of D = 0.5 h^{-1}, T = 30 °C, and 250 rpm. Mean DAPI-FI (blue) at each time point was calculated as the average DAPI-FI of all events within the cell gate. **A:** *Bradyrhizobium* sp. **B:** *E. coli* and **C:** *S. rhizophila*.

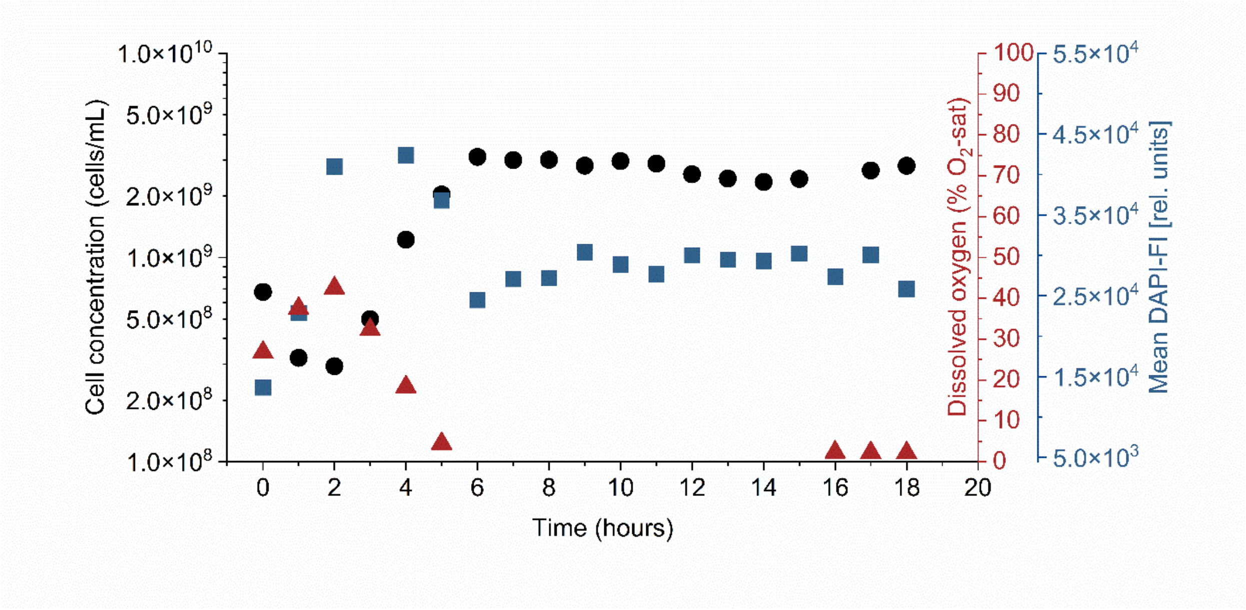

Figure SI 2. Automatic on-line flow cytometric monitoring of cell growth in continuous bioreactors for *E. coli* with D = 0.31 h^{-1}. Cell concentration (black) and dissolved oxygen concentration (red) were measured hourly at a dilution rate of D = 0.31 h^{-1}, T = 30 °C, and 250 rpm. Mean DAPI-FI (blue) at each time point was calculated as the average DAPI-FI of all events within the cell gate.

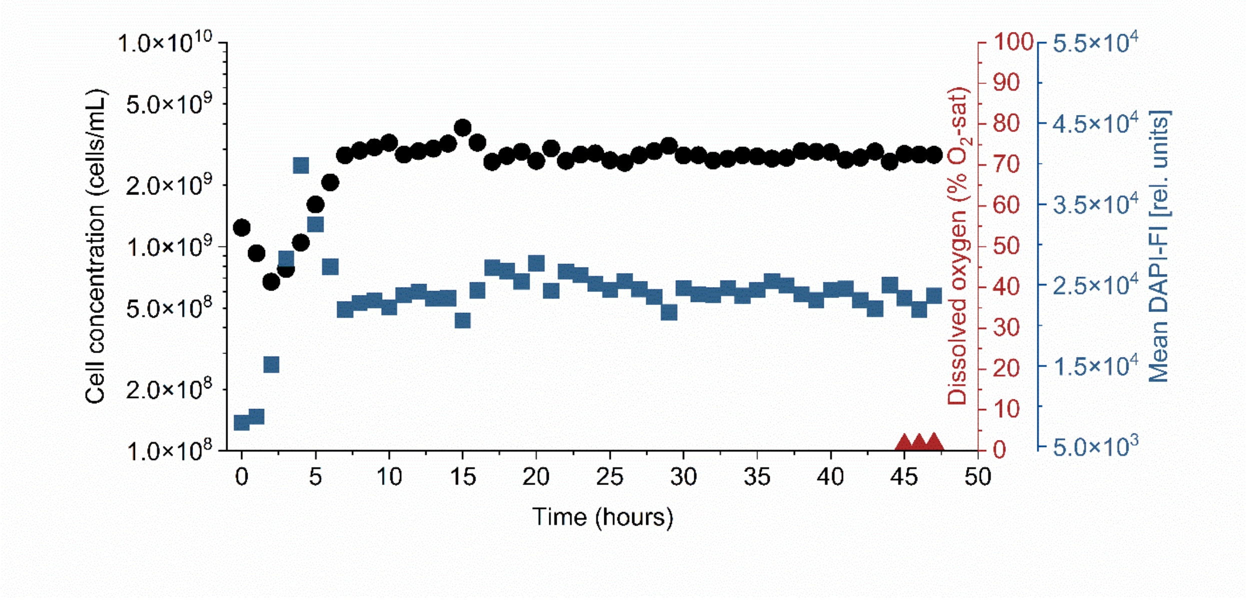

Figure SI 3. Automatic on-line flow cytometric monitoring of cell growth in continuous bioreactors for *E. coli* with D = 0.19 h^{-1}. Cell concentration (black) and dissolved oxygen concentration (red) were measured hourly at a dilution rate of D = 0.19 h^{-1}, T = 30 °C, and 250 rpm. Mean DAPI-FI (blue) at each time point was calculated as the average DAPI-FI of all events within the cell gate.

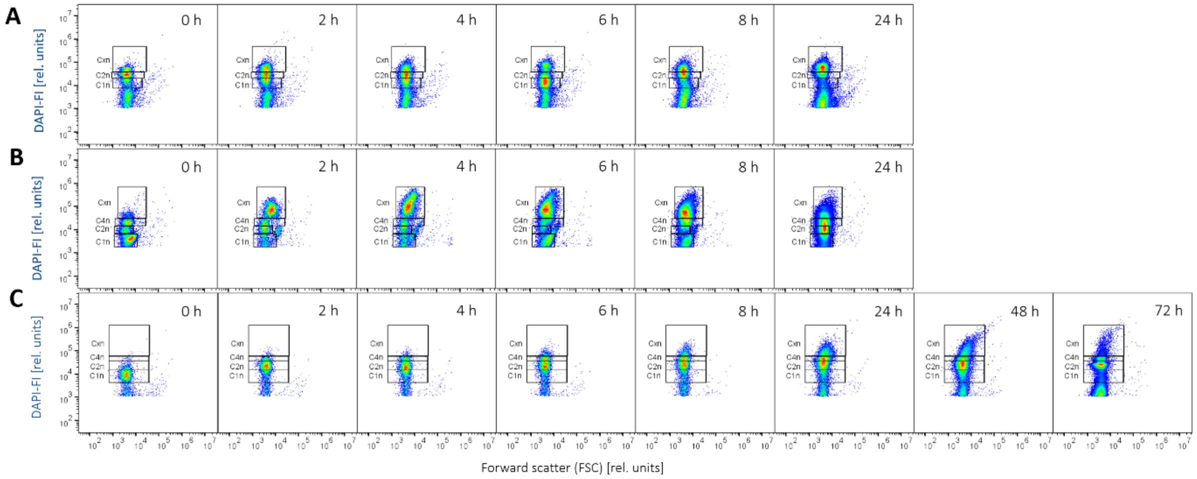

Figure SI 4. Subpopulation gating of DAPI-FI [rel. units] versus forward scatter (FSC) [rel. units] in batch-cultivated strains. Rows A–C show *Bradyrhizobium* sp., *E. coli* and *S. rhizophila*, respectively. DAPI-positive cells were subdivided into subpopulations with approximately one, two, four or more chromosome equivalents (C1n, C2n, C4n, Cxn); for *Bradyrhizobium* sp. and *E. coli* only C1n, C2n and Cxn were resolved, whereas all four subpopulations were distinguished for *S. rhizophila*. Columns correspond to time points 0, 2, 4, 6, 8 and 24 h for *Bradyrhizobium* sp. and *E. coli*, and 0, 2, 4, 6, 8, 24, 48 and 72 h for *S. rhizophila*. Samples correspond to the batch experiments associated with Figure 3. **A**: *Bradyrhizobium* sp., **B**: *E. coli* and **C**: *S. rhizophila*.

Supplementary Figures 5-7

Due to DAPI staining and its relatively high resolution, bacterial subpopulations can be distinguished based on their DNA content. These subpopulations differ in chromosome number, allowing them to be categorized accordingly. Once identified, they are gated, and their proportions relative to the total cell population are calculated. These proportions are then visualized using a barcode plot generated by the flowCyBar bioinformatics tool[1]. This visualization enables to track the emergence of subpopulation distributions over time. It also allows to determine whether the bacterial population is shifting toward subpopulations with a single chromosome or those with multiple chromosomes. From this data, we can infer whether the bacterial population is primarily engaged in DNA synthesis or cell division.

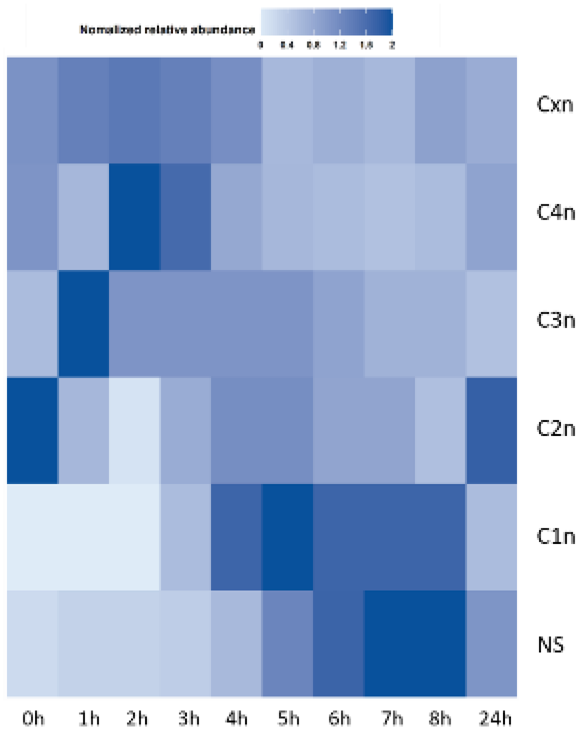

Figure SI 5. Barcode plot illustrating the distribution of *Bradyrhizobium* sp. subpopulations with varying chromosome numbers during batch cultivation, as described in Section 2.2. Cells were fixed using PFA/EtOH and stained with DAPI staining solution (0.24 µM, section 2.4). Distinct subpopulations were identified: C1n (cells with one chromosome number), C2n (two chromosome numbers), C3n and C4n (three and four chromosome numbers, respectively), and Cxn (cells containing more than four chromosome numbers). NS denotes cells that were either unstained or incompletely stained by DAPI. Increasingly darker shades of blue indicate higher relative abundance within each subpopulation. The plot was generated using the flow cytometry visualization tool flowCyBar.

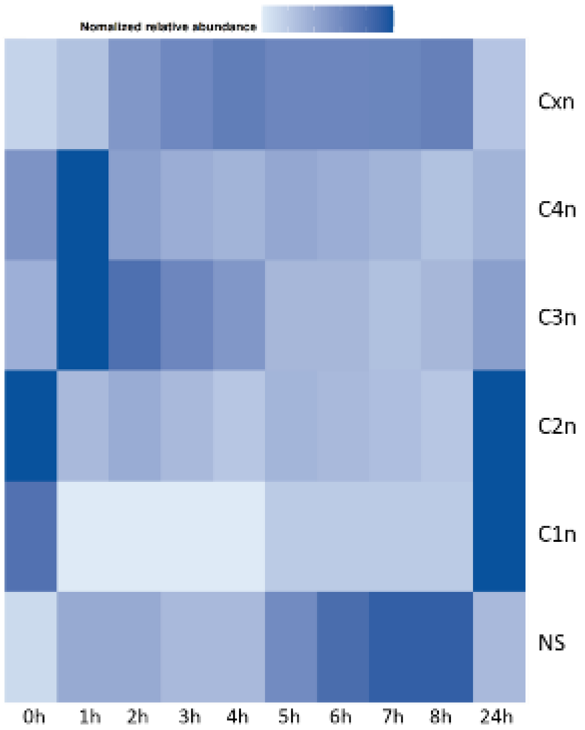

Figure SI 6. Barcode plot illustrating the distribution of *E. coli* subpopulations with varying chromosome numbers during batch cultivation, as described in Section 2.2. Cells were fixed using PFA/EtOH and stained with DAPI staining solution (0.24 µM). Distinct subpopulations were identified: C1n (cells with one chromosome number), C2n (two chromosome numbers), C3n and C4n (three and four chromosome numbers, respectively), and Cxn (cells containing more than four chromosome numbers). NS denotes cells that were either unstained or incompletely stained by DAPI. Increasingly darker shades of blue indicate higher relative abundance within each subpopulation. The plot was generated using the flow cytometry visualization tool flowCyBar.

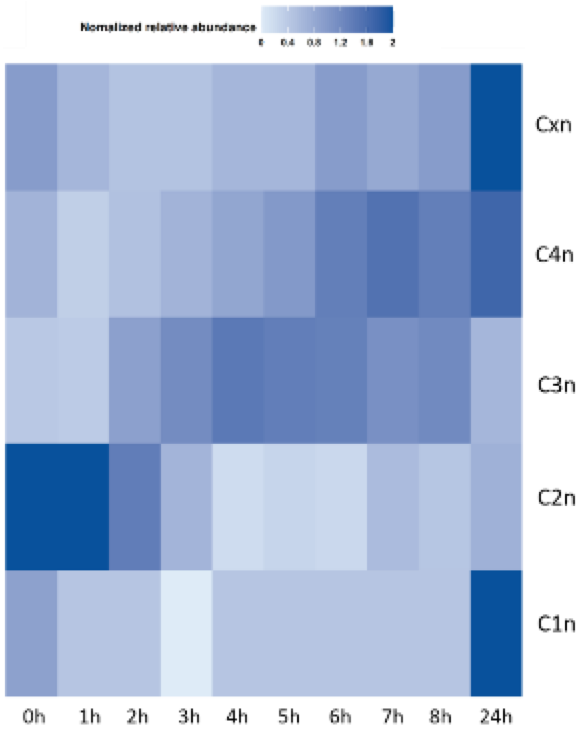

Figure SI 7. Barcode plot illustrating the distribution of *S. rhizophila* subpopulations with varying chromosome numbers during batch cultivation, as described in Section 2.2. Cells were fixed using PFA/EtOH and stained with DAPI staining solution (0.24 µM). C1n (cells with one chromosome number), C2n (two chromosome numbers), C3n and C4n (three and four chromosome numbers, respectively), and Cxn (cells containing more than four chromosome numbers). Increasingly darker shades of blue indicate higher relative abundance within each subpopulation. The plot was generated using the flow cytometry visualization tool flowCyBar.

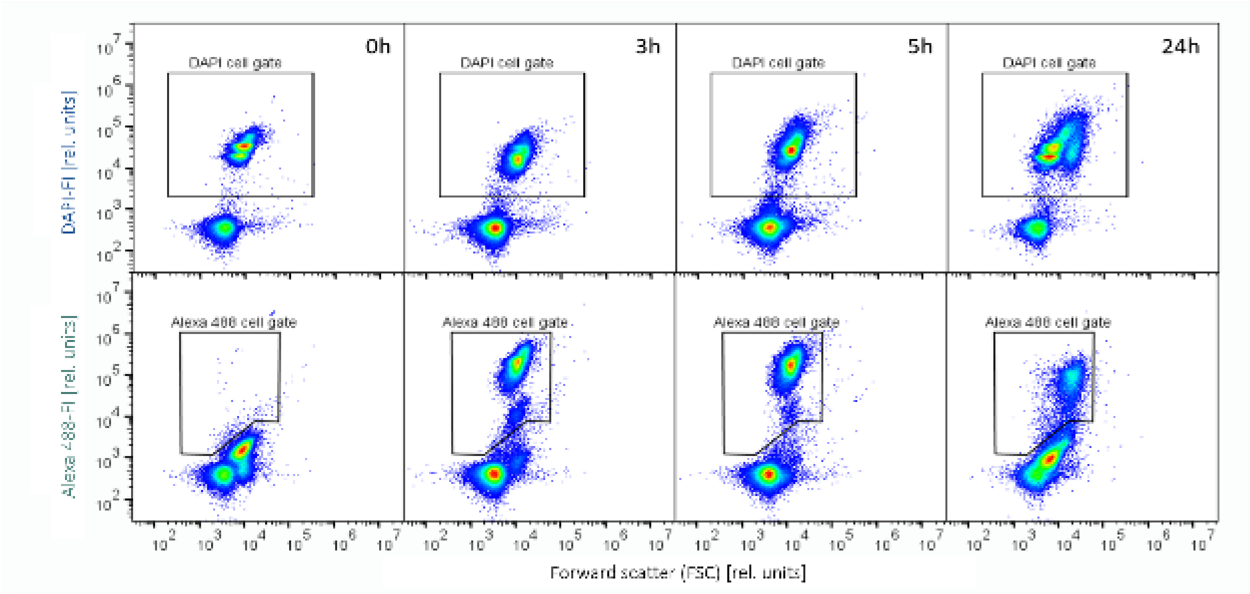

Figure SI 8. 2D flow cytometric plots of an *E. coli* culture sampled at various time points. The cells were batch-cultivated in a 24-well plate, manually sampled, and processed. Following fixation using the standard PFA/EtOH protocol[2], the cells were double-stained with DAPI and Alexa 488.

- *Bradyrhizobium* sp. (obtained from Schlechter et al. [3])
- *Cupriavidus necator* (DSM 13513)
- *Escherichia coli* K-12 LE392 (DSM 4230)
- *Kocuria rhizophila* (DSM 348)
- *Mycobacterium rhodesianum* (MB126)
- *Pseudomonas citronellolis* (obtained from Schlechter et al. [3])
- *Paenibacillus polymyxa* (DSM 36)
- *Pseudomonas putida* KT2440
- *Sphingomonas melonis* (obtained from Schlechter et al. [3])
- *Stenotrophomonas rhizophila* (DSM 14405)

List SI 1. List of bacterial strains tested for the Alexa 488 and DAPI double staining procedure.

References

1. Koch, C., Harnisch, F., Schröder, U. & Müller, S. Cytometric fingerprints: evaluation of new tools for analyzing microbial community dynamics. *Frontiers in Microbiology* **5**, 273 (2014).

2. Cichocki, N. *et al.* Bacterial mock communities as standards for reproducible cytometric microbiome analysis. *Nature Protocols* **15**, 2788–2812 (2020).

3. Schlechter, R. O. *et al.* Chromatic bacteria – A broad host-range plasmid and chromosomal insertion toolbox for fluorescent protein expression in bacteria. *Frontiers in Microbiology* **9**, 3052 (2018).
